# Multilevel analysis of the determinants of the global assessment of functioning in an inpatient population

**DOI:** 10.1186/1471-244X-14-63

**Published:** 2014-03-04

**Authors:** Karen A Urbanoski, Cecile Henderson, Saulo Castel

**Affiliations:** 1 Social and Epidemiological Research, Centre for Addiction and Mental Health, T317, 33 Russell St., Toronto, ON M5S 2S1, Canada; 2 Dalla Lana School of Public Health, University of Toronto, Toronto, Canada; 3 NHS Lothian, Edinburgh, UK; 4 Department of Psychiatry, Sunnybrook Health Sciences Centre, Toronto, Canada; 5 Department of Psychiatry, University of Toronto, Toronto, Canada

**Keywords:** Global assessment of functioning, Inpatient care, Multilevel modeling, Clinical assessment

## Abstract

**Background:**

The Global Assessment of Functioning (GAF) is a widely used measure of psychiatric symptoms and functioning, yet numerous concerns persist about its reliability and validity. The objective of this study was to determine the extent to which GAF scores reflect physician-related differences in addition to information about patients.

**Methods:**

This is a secondary analysis of clinical data collected between 2005 and 2010 from inpatients at a psychiatric hospital (N = 1,852). Multilevel modeling was used to estimate the influence of physicians on GAF scores at admission and on the change between admission and discharge, controlling for patient clinical presentation.

**Results:**

Controlling for patient-level predictors, 7% of the residual variance in admission GAF scores and 8% of the residual variance in change scores was at the physician level. The physician-level variance was significantly larger than zero in both models.

**Conclusions:**

Although statistically significant, estimates of physician-level variance were not overwhelming, suggesting that the GAF was rated in a consistent manner across physicians in this hospital. While results lend support to the utility of the GAF for drawing comparisons between patients seen by different physicians across a large institution, further study is necessary to determine generalizability and to assess differences across multiple institutions.

## Background

The Global Assessment of Functioning (GAF) provides a global rating of clinical severity across psychiatric diagnoses [1]. It is well-known internationally, available in many languages, and used widely as a measure of psychiatric symptom severity and functioning [2-4]. There are many reasons for its popularity. It ensures that not only symptom severity but also social and occupational functioning is included in the clinical assessment [5]. By incorporating both school and work dimensions of functioning, it applies to a wide range of ages. As a single rating, it is easy to administer, relatively inexpensive, and intuitively and analytically appealing [3,6]. It is not surprising that, in a review of the literature published from 1990–2002, the GAF was among the most widely used outcome measures in psychiatric research [6].

Despite its popularity, numerous concerns persist about the GAF, including its reliability and validity, and the level of subjectivity in the rating process [2,7]. Low inter-rater reliability has been reported in routine clinical settings [8]. Brief training can improve reliability, although the duration of the improvement is unclear [9,10]. Patient-level analyses have consistently identified symptom severity as the most important determinant of GAF scores, with smaller contributions made by measures of social and occupational functioning [11-15]. There is evidence, however, that factors other than patient presentation also predict GAF scores, including psychiatrist gender and years of practice [16] and the site of treatment [17]. Although providing preliminary evidence of potential bias in GAF scores, these studies did not take into account the clustering of patients of particular types within providers and programs, or the multilevel nature of the information that is collected during routine clinical practice. This is a key concern for a measure such as the GAF, which is criticised for a perceived high level of subjectivity in the rating process.

The appropriateness of the GAF as a measure of patient outcome and program performance rests on the assumption that individual clinical presentation determines the score. There is little empirical data available to support this assumption. Particularly if the GAF is to be used for performance measurement, program comparisons, and resource allocation [2], it is imperative that influences other than clinical presentation are identified and investigated. The primary aim of this study was to determine the extent to which GAF scores reflect only information about patients or whether they also reflect physician-related differences. To date, no prior studies have made use of the natural clustering of patients within physicians or units to evaluate predictors of the GAF, or partitioned the variance in scores to patient versus these higher levels.

## Methods

### Study sample and procedures

We analysed administrative data from inpatient clinical assessments conducted in a single psychiatric hospital over a 4.5-year period (October 2005-March 2010, N = 1,852). The hospital is located in a densely populated suburban region in Ontario, Canada. The hospital’s 320 beds are housed in four main programs: 1) a general psychiatry program for adults (18+ years old); 2) a forensics program; 3) a program for young adults (18–30 years old) and those with psychiatric and developmental disorders; and 4) a program with wards specific for geriatric psychiatry and acquired brain injury. Each program contained multiple units, to which patients were assigned based on diagnosis, chronicity and/or severity of illness. Within units, patient assignment to physicians was reportedly done based on physician availability, but was random according to patient diagnosis and clinical presentation. The study was approved by the Research Ethics Board at Ontario Shores Centre for Mental Health Sciences.

Data from admission and discharge assessments were abstracted from a centralized hospital database that had been de-identified for this project. As a secondary analysis of a de-identified administrative dataset, consent was not obtained from individual patients. Anonymous unique identifiers were used to identify patients, episodes of care within patients, attending physicians, and hospital unit. For patients with multiple episodes of care during the study period, we selected the first episode for analysis. The analytical sample represents all patients admitted for inpatient care at the hospital over the 4.5 years, with the exception of a small number with outlying values for age (removed for confidentiality concerns). Most patients (76.4%) had only one episode of care during this time (maximum = 11, mean = 1.4). Two thirds (67.6%) were male, and average age at admission was 43.5 years old (SD = 18.6), ranging from 17 to 95 years old. The most common diagnosis was schizophrenia (67.6%), followed by mood disorders (15.7%) and dementia (12.7%). The median length of stay in hospital was 61 days (SD = 217, ranging from 0–1584 days). The dataset included 47 physicians and 14 units. The number of physicians per unit ranged from 2 to 24 (median = 6), and 51% of physicians worked on more than one unit during the study period. The number of patients seen by each physician ranged from 1 to 171 (mean = 39.4).

Of the 1,852 patients admitted, data from the corresponding discharge assessment were missing for 215 patients (11.6%). These missing records involved unplanned discharges, hospitalizations of brief duration (<72 hours), and GAF ratings of 0, indicating insufficient information with which to make a rating. In addition, 556 patients (30.0%) had their GAF ratings made by different physicians and/or on different units at admission and discharge. A change score, reflecting change in the GAF during a single episode of care, was calculated for patients with corresponding admission and discharge assessments, conducted by the same physician in the same setting (N = 1,081). This subset of the data included 41 physicians and 14 units. The number of physicians per unit ranged from 1 to 16 (median = 3), and 37% of physicians worked on more than one unit during the study period. The number of patients seen by each physician ranged from 1 to 136 (mean = 26.4).

### Measures

The GAF provides a single dimensional rating of social, psychological, and occupational functioning [1]. Scores range from 1 to 100, with 100 representing an absence of symptoms and superior functioning. Guidelines for rating the GAF describe symptoms and levels of functioning in 10-point intervals, with brief explanations and examples. At the study site, the GAF is scored at admission and discharge by physicians as part of the routine clinical assessment. Physicians at this hospital received a 1-hour training on the use of the GAF.

Activities of Daily Living (ADL) were assessed with two scales from the Resident Assessment Instrument-Mental Health (RAI-MH) [18]. Both scales are rated by nurses at admission and discharge. The ADL Short Form contains 5 items on level of impairment with respect to personal hygiene, walking, toilet use, and eating in the past 3 days [19]. Total scores range from 0–20, with higher scores indicating greater ADL impairment. The Instrumental ADL (IADL) scale contains an additional 5 items on level of impairment with respect to meal preparation, managing medications and finances, transportation, and telephone use. Total scores range from 0–30, with higher scores indicating greater impairment.

Patient gender, age at admission, and primary Axis I diagnosis were also abstracted from the centralized database.

### Analysis

Preliminary analyses examined the bivariate associations between the admission GAF ratings and other patient-level variables (i.e., gender, age, diagnosis, ADL and IADL). We also ran a standard linear regression to identify the independent patient-level predictors of admission GAF scores. A multilevel model was then used to estimate the influence of physicians on GAF scores. GAF scores were approximately normally distributed and modeled using maximum likelihood estimation. A three-level model, specifying units, physicians, and patients, was required to adequately account for the nested data structure. We fit random intercepts for unit and physician, allowing for cross-classification to accommodate physicians who worked on more than one unit [20]. The random intercept for unit was included to minimize the chances of attributing patient-level variance to physicians, given that various aspects of patient clinical presentation determined the unit of care. We used the estimates of unit-, physician- and patient-level variance in GAF scores to calculate two intraclass correlations (ρ) quantifying the proportion of variance in GAF scores at the physician level, and the proportion at the physician and unit levels combined [20]. The model was repeated with the change in GAF score between admission and discharge as the dependent variable. Patient-level predictors in this model included gender, age at discharge, diagnosis, and during-treatment change in ADL and IADL. Analyses were conducted in Stata 12.0 and used an alpha level of .05.

## Results

Across the full sample, GAF scores averaged 36.3 at admission (SD = 13.0). Scores were slightly lower among women (mean = 34.6, SD = 11.8) than men (mean = 37.2, SD = 13.5; t = −4.04, df = 1850, p < .001). Scores were lowest for those with a diagnosis of dementia (mean = 21.7, SD = 12.2), relative to those with schizophrenia (mean = 37.7, SD = 10.8), mood disorders (mean = 38.4, SD = 13.0) or other diagnoses (mean = 42.2, SD = 13.4; F = 149.57, df = 3,1848, p < .001), and were inversely associated with age and the measures of daily functioning at admission (Table 1). All of these patient-level factors were independently associated with admission GAF scores in a standard linear regression model (Table 2).

**Table 1:** Spearman correlations between GAF scores at admission and patient-level predictors (N = 1,852)*

	**Mean(SD)**	**Min-Max**	**1.**	**2.**	**3.**	**4.**
1. GAF	36.3(13.0)	2-80	1.00			
2. Age	43.5(18.6)	17-95	−0.30	1.00		
3. ADL-short	1.8(4.1)	0-20	−0.44	0.43	1.00	
4. IADL	10.8(11.1)	0-30	−0.41	0.42	0.67	1.00

*All correlations p < .001.

(I)ADL = (Instrumental) Activities of Daily Living.

**Table 2:** Patient-level predictors of admission GAF scores (N = 1,852)

	**Model: F = 108.90, df = 7, 1844, p < .001**			
**Independent variable**	**est**	**se**	**t**	**p**
Intercept	40.48	0.91	44.30	<.001
Male gender	1.49	0.57	2.63	.009
Age	−0.04	0.02	−2.16	.031
Diagnosis				
Schizophrenia	---	---	---	
Dementia	−5.13	1.11	−4.63	<.001
Mood	2.52	0.74	3.40	.001
Other	5.48	0.82	6.71	<.001
ADL	−0.58	0.09	−6.46	<.001
IADL	−0.26	0.03	−7.94	<.001

Controlling for patient-level predictors, only 7% of the residual variance in admission GAF scores was at the physician level, while 29% was accounted for by physicians and units combined (Table 3). A likelihood ratio test comparing the 3-level model to a 2-level model excluding the random intercept for physician (i.e., accounting only for the nesting of patients in units) indicated that the physician-level variance was significantly larger than zero (χ^2^ = 64.88, p < .001).

**Table 3:** Estimating provider-level variance in GAF scores at admission (N = 1852)

	**Model: χ**^**2**^ **= 222.74, df = 7, p < .001**			
**Parameter**	**est**	**se**	**z**	**p**
Intercept	40.25	1.97	20.45	<.001
Male gender	0.45	0.54	0.83	.404
Age	0.03	0.02	1.24	.215
Diagnosis				
Schizophrenia	---	---	---	
Dementia	−2.71	1.21	−2.23	.026
Mood	3.72	0.70	5.28	<.001
Other	5.30	0.80	6.65	<.001
ADL	−0.45	0.09	−5.23	<.001
IADL	−0.25	0.04	−6.82	<.001
**Variance:**				
Unit-level	30.25	14.31		
Physician-level	10.39	3.79		
Individual-level	100.48	3.35		
ρ(physician)*	0.07			
ρ(physician, unit)^†^	0.29			

*Intraclass correlation within physicians: physician-level variance/total variance.

^†^Intraclass correlation within units and physicians: (unit-level variance + physician-level variance)/total variance.

GAF scores increased by an average of 7.4 (SD = 11.8) between admission and discharge. Controlling for patient-level predictors, 8% of the residual variance in GAF change scores was at the physician level and 9% was accounted for by physicians and units combined (Table 4). Again, the likelihood ratio test comparing the model to one excluding the random intercept for physician indicated that the physician-level variance was significantly larger than zero (χ^2^ = 28.77, p < .001).

**Table 4:** Estimating physician-level variance in the during-treatment change in GAF scores (N = 1,081)

	**Model: χ**^**2**^ **= 32.91, df = 7, p < .001**			
**Parameter**	**est**	**se**	**z**	**p**
Intercept	7.56	1.63	4.64	<.001
Male gender	−0.62	0.77	−0.80	.424
Age(at discharge)	0.02	0.03	0.64	.519
Diagnosis				
Schizophrenia	---	---	---	
Dementia	−4.18	1.48	−2.82	.005
Mood	2.37	1.00	2.39	.017
Other	−0.89	1.04	−0.85	.395
Change in ADL	−0.49	0.19	−2.61	.009
Change in IADL	−0.03	0.07	−0.39	.699
**Variance:**				
Unit-level	1.21	1.64		
Physician-level	11.35	4.50		
Individual-level	122.91	5.40		
ρ(physician)*	0.08			
ρ(physician, unit)^†^	0.09			

*Intraclass correlation within physicians: physician-level variance/total variance.

^†^Intraclass correlation within units and physicians: (unit-level variance + physician-level variance)/total variance.

## Discussion

This study provides an important look at the extent of physician influences on patients’ GAF scores in a large cohort of psychiatric inpatients. As expected, patient-level factors including older age, greater impairment in activities of daily living, and a diagnosis of dementia predicted lower GAF scores at admission to hospital. Although statistically significant, the gender difference in GAF scores was slight and not clinically meaningful. Most relevant to the present study, however, the proportion of variance in admission GAF scores that was attributed to physicians rather than to differences in patients’ clinical presentation was fairly low at 7%. Similarly, 8% of the variance in GAF change scores from admission to discharge was at the physician level. Although statistically significant, these estimates of variance are not overwhelming, and appear to signal that, in this hospital at least, there was minimal contribution of physician influences to GAF scores.

Although the focus on the routine performance of the GAF in a real-world clinical setting is a strength of the present study, the task of estimating the variance in GAF scores accounted for by physicians was complicated by the fact that patients were not randomized to physicians. In a hospital setting, a physician may see a particular type of patient as a result of their affiliation with one or more units that serves a particular clientele. With patients assigned to units based on clinical and other personal factors, similarities in scores on the GAF and other assessment tools rated by the same physician can legitimately result from patient characteristics. To the extent that patient-level predictors of GAF scores are missing from the model, our estimate of provider-level variance may be an exaggeration [21]. In addition to controlling for a number of patient characteristics that may account for differences in GAF scores, we addressed the lack of randomization of patients to physicians by including the unit of care as a random factor in the model. In so doing, we aimed to capture additional variability resulting from the sorting of patients of particular types into units and, therefore, to their affiliated physicians. To some degree, the estimate of unit-level variance may also reflect shared assessment and scoring practices that develop within units^a^. The proportion of variance attributed to physicians and units combined (ρ(physician, unit) in Table 3), reflects the correlation between patients seen by the same physician on the same unit [20]. That is, within a given unit in the hospital, there appears to be a non-trivial proportion of variance in admission GAF scores (29%) that is shared between patients seen by the same physician. However, this figure falls to 7% when the variance attributable to unit is partitioned out (ρ(physician) in Table 3). This proportion of variance at the provider level reflects the correlation between patients seen by the same physician on different units [20], and is likely a more accurate reflection of the extent to which there are physician-related differences in GAF scores (i.e., independent of patient clinical presentation). The unit-level variance itself likely reflects a mix of variability resulting from unmodelled patient factors, as well as shared assessment and scoring practices based on unit characteristics and circumstances.

In the model predicting patient-level change in GAF scores between admission and discharge, there is little difference between the intraclass correlation estimates when unit level variance is included. It is possible that the initial rating at admission may provide a benchmark against which the second rating is made, such that unmodelled patient factors and the process by which they are assigned to units in the hospital accounts for less of the variance in GAF change scores, relative to scores at admission. It should be noted that, other than physician influences on the way that the GAF is scored, physician-level variance in patient improvement on the GAF could also reflect differences in physician effectiveness. It is a limitation of the present study that we were unable to determine the sources of physician-level variance. Providing an important extension to this work, datasets that include the characteristics of physicians may be valuable in terms of examining more specifically whether, and how, physician-related factors influence GAF scores.

A final limitation of this study relates to its observational nature. Randomizing patients to physicians, and having multiple physicians rate the same randomly-assigned patient, would offer a stronger test of physician-level influences on GAF scores. That said, the broad coverage of the data, capturing a complete cohort of people receiving inpatient treatment at a hospital over a 4.5-year period, and the investigation of the GAF as it is rated in routine clinical practice are important strengths of this work.

## Conclusions

The ideal measure of symptom severity and functioning would be sensitive to individual clinical presentation alone. Findings from the present study suggest that GAF scores at this institution appeared to be minimally influenced by physicians, once patient characteristics were taken into account. In other words, the GAF appeared to be rated in a fairly consistent manner across physicians. These results lend support to the utility of the GAF for drawing comparisons between patients seen by different physicians across a large institution serving a heterogeneous clientele. This is an important insight given the popularity of the GAF in evaluating outcomes and its potential utility for case-mix adjustment in resource allocation. This work sets the stage and highlights the need for studies of wider scope, evaluating the multilevel determinants of the GAF and other similar measures across broader systems of care.

## Endnote

^a^We thank the reviewer for raising this point.

## Abbreviations

GAF: Global assessment of functioning; ADL: Activities of daily living; RAI-MH: Resident assessment instrument-mental Health; SD: Standard deviation.

## Competing interests

The authors declare that they have no competing interests.

## Authors’ contributions

SC conceived of the study, and he and CH arranged for and cleaned the administrative data. KU designed and conducted the analysis. All authors were involved in interpreting the findings. KU drafted the manuscript, and all authors contributed to revising and editing. All authors have reviewed and approved the final manuscript.

## Pre-publication history

The pre-publication history for this paper can be accessed here:

http://www.biomedcentral.com/1471-244X/14/63/prepub
